# Predictors and predictive effects of acute pain trajectories after gastrointestinal surgery

**DOI:** 10.1038/s41598-022-10504-5

**Published:** 2022-04-20

**Authors:** Qing-Ren Liu, Yu-Chen Dai, Mu-Huo Ji, Li-Li Qiu, Pan-Miao Liu, Xing-Bing Sun, Jian-Jun Yang

**Affiliations:** 1 Department of Anesthesiology, Xishan People’s Hospital of Wuxi City, Wuxi, 214105 China; 2 Department of Anesthesiology, Zhongda Hospital, Medical School, Southeast University, Nanjing, 210009 China; 3 Department of Anesthesiology, The Second Affiliated Hospital, Nanjing Medical University, Nanjing, 210011 China; 4 Department of Anesthesiology, Pain and Perioperative Medicine, The First Affiliated Hospital of Zhengzhou University, NO. 1 East Jianshe Road, Zhengzhou, 450000 China

**Keywords:** Clinical trial design, Outcomes research, Gastroenterology, Risk factors

## Abstract

Few studies have investigated factors associated with acute postsurgical pain (APSP) trajectories, and whether the APSP trajectory can predict chronic postsurgical pain (CPSP) remains unclear. We aimed to identify the predictors of APSP trajectories in patients undergoing gastrointestinal surgery. Moreover, we hypothesised that APSP trajectories were independently associated with CPSP. We conducted a prospective cohort study of 282 patients undergoing gastrointestinal surgery to describe APSP trajectories. Psychological questionnaires were administered 1 day before surgery. Meanwhile, demographic characteristics and perioperative data were collected. Average pain intensity during the first 7 days after surgery was assessed by a numeric rating scale (NRS). Persistent pain intensity was evaluated at 3 and 6 months postoperatively by phone call interview. CPSP was defined as pain at the incision site or surrounding areas of surgery with a pain NRS score ≥ 1 at rest. The intercept and slope were calculated by linear regression using the least squares method. The predictors for the APSP trajectory and CPSP were determined using multiple linear regression and multivariate logistic regression, respectively. Body mass index, morphine milligram equivalent (MME) consumption, preoperative chronic pain and anxiety were predictors of the APSP trajectory intercept. Moreover, MME consumption and preoperative anxiety could independently predict the APSP trajectory slope. The incidence of CPSP at 3 and 6 months was 30.58% and 16.42% respectively. APSP trajectory and age were predictors of CPSP 3 months postoperatively, while female sex and preoperative anxiety were predictive factors of CPSP 6 months postoperatively. Preoperative anxiety and postoperative analgesic consumption can predict APSP trajectory. In addition, pain trajectory was associated with CPSP. Clinicians need to stay alert for these predictors and pay close attention to pain resolution.

## Introduction

Pain management continuously faces major challenges, although acute postsurgical pain (APSP) has attracted increased attention^1^. A larger cross-sectional study with 15,000 patients showed that 37% experienced moderate pain and 11% experienced severe pain within the first 24 h post-operation^2^. Moderate or severe APSP has an adverse effect on early recovery. The consequences of poorly controlled APSP include prolonged hospital length of stay and increased opioid consumption and medical expenses^3^. Moreover, APSP is also associated with some major poor outcomes, such as postoperative delirium, cardiovascular events, thromboembolism, and pulmonary complications^4–6^. Persistent acute pain can transition to chronic pain which may lead to long-term disability, decreased quality of life, and increased medical burden^1,7,8^.

Effective pain management depends on accurate pain assessment. A single pain score using current tools, such as the numerical rating scale (NRS) and visual analogue scale (VAS) is too imprecise to reflect the complexity of pain multidimensionally^9^. Chapman et al.^10^ developed a more precise tool for pain measurement called the acute pain trajectory, which can evaluate the effect of pain control dynamically and accurately, where initial pain intensity is characterised through the intercept while the resolution of pain is presented by the slope. The acute pain trajectory is advantageous in individualised pain treatment for postoperative patients since it focuses more on rapid pain resolution, rather than a reduction in pain intensity.

Chronic postsurgical pain (CPSP) refers to pain that occurs in the surgical incision or surrounding areas continuously or intermittently for at least 3 months. Other causes, such as continuing malignancy, chronic infection or continuation of chronic preoperative pain, for the pain should be excluded. Inadequate acute pain control can develop into a poor acute pain trajectory with a positive or flat slope, which is significantly correlated with CPSP^11^. Furthermore, poor pain trajectory after trauma is also related to posttraumatic stress disorder, persistent depression and cognitive dysfunction^12^. Given individual differences in the development of chronic pain, early identification of and intervention for major risk factors for CPSP will contribute to reducing the incidence of CPSP^13^.

Recently, studies have explored the risk factors for chronic pain after gastrointestinal surgery. Some predictors such as young age, female sex, preoperative chronic pain, and preoperative anxiety and depression have been shown to independently predict the CPSP. However, there are limited studies exploring the acute pain trajectory after gastrointestinal surgery. Chang et al.^14^ described the APSP trajectory for colorectal cancer surgery and explored the relationship between the APSP trajectory and postoperative outcomes such as tumour recurrence and mortality. Nevertheless, no studies have investigated the association between APSP trajectories and CPSP.

This study aims to identify the risk factors for acute pain trajectory after gastrointestinal surgery by investigating demographic, psychological and clinical variables, and to explore the predictive effect of APSP trajectories on CPSP. We hypothesised that MME consumption and some psychological factors were independently associated with APSP trajectories and poor pain trajectories could predict the development of CPSP.

## Methods

### Study design and participants

This prospective cohort study protocol was registered at the Chinese Clinical Trial Registry (ChiCTR1900024837). Patients for elective gastrointestinal surgery due to gastric or colorectal disease were enrolled between August 2019 and January 2020 at Zhongda Hospital, Southeast University. The study was approved by the Institutional Ethics Committee for Clinical Research of Zhongda Hospital, affiliated to Southeast University (No. 2019ZDSYLL084-P01). All methods were performed in accordance with the relevant guidelines and regulations. We recruited 345 patients to investigate the risk factors for acute pain trajectory after gastrointestinal surgery and explore the predictive effect of APSP trajectories on CPSP. Written informed consent was obtained as a condition to participate in the study. The inclusion criteria were as follows: (1) patients undergoing elective gastrointestinal surgery under general anaesthesia, (2) age ≥ 18 years, (3) physical status classification of the American Society of Anaesthesiologists (ASA) I to III, and (4) without signs indicative of severity. The excluded criteria included patients with expressive language disorder, hearing impairment or visual impairment.

### Demographic characteristics and psychological questionnaires

On the day before surgery, demographic data including gender, age, height, weight, occupation status, education level, smoking (never, former, current), alcohol drinking (never, former, current), hypertension, diabetes, coronary heart disease, physical status classification of the ASA, history of previous chronic pain and previous surgery were collected. Moreover, anxiety, depression and fear of surgical procedures were measured using the Hospital Anxiety and Depression Scale (HADS) and Surgical Fear Questionnaire (SFQ) respectively, and expected postsurgical pain intensity was also assessed.

Participants who suffered from pain for more than 3 months were classified as having previous chronic pain. Meanwhile, whether the patients had a history of surgery was recorded. The HADS^15^, consisting of two seven-item subscales, has been used to measure anxiety and depression levels among patients in nonpsychiatric hospital settings. Subscale scores range between 0 and 21, with higher scores representing higher levels of anxiety and depression. The SFQ^16^, a valid and reliable indicator of surgical fear, consists of two eight-item subscales: fear of the short-term consequences of surgery (SFQ-s) and fear of the long-term consequences (SFQ-l). Each item has a score range of 0–10. Expected postsurgical pain was measured with an 11-point numerical rating scale (NRS) (on a scale from 0 = no pain at all to 10 = strongest pain imaginable)^17^.

### Surgery and anaesthesia

All surgeries were performed according to the standard protocol by 3 surgeons. Information on the type of surgery, surgical site, duration of surgery, number of drainage tubes, and length of hospital stay was collected. In addition, preoperative clinical factors were obtained from the electronic medical record system. Surgical sites included the stomach, colorectum, and small bowel. The type of surgery was open or laparoscopic.

All patients had no preoperative medications. After admission to the operating room, a peripheral intravenous catheter was inserted. Parameters such as ART, ECG, SpO_2_, and P_ET_CO_2_ were monitored routinely. A standardised anaesthesia protocol was administered by one of the anaesthesiologists from the study team. General anaesthesia was rapidly induced with intravenous sufentanil 0.3 μg/kg, propofol 2–2.5 mg/kg, and rocuronium 0.6 mg/kg. After intubation, all patients were placed on the mechanical ventilator with tidal volume (VT) 8–10 ml/kg and respiratory rate (RR) 10–12 times/min. General anesthesia was maintained with inhalation of 2.0–3.0% sevoflurane (0.8–1.0 MAC) and intravenous pumping of remifentanil 0.1–0.3 μg/kg/min and intravenous injection of cisatracurium. Sevoflurane and remifentanil were discontinued at the time of skin suturing, and additional sufentanil 0.1 μg/kg was administered intravenously.

### Postoperative analgesia

The primary outcome was average pain intensity assessed using pain NRS scores from 1 to 7 days after surgery. Patients were asked to report their level of pain during movement (changing position from supine to either sitting or standing) in the past day. The average pain intensity over 7 days post-operation was continuously recorded. Patients were questioned about their pain intensity by phone 3 and 6 months postoperatively. CPSP was defined as pain at the incision site or surrounding areas of surgery with pain NRS score ≥ 1 at rest.

Patients received routine postoperative pain management. In the postanaesthesia care unit (PACU), tramadol 50 mg or oxycodone 2 mg was administered intravenously when the NRS pain score at rest was ˃ 3. On the ward, patients received oral celecoxib 200 mg every 12 h and patient-controlled intravenous analgesia (PCIA) with oxycodone or sufentanil. Additionally, during the period of self-controlled analgesia, rescue analgesia was provided with intravenous tramadol if necessary. Analgesic consumption during the first 24 h postoperatively was recorded from the time patients arrived in the PACU. To compare analgesic consumption, intravenous opioid analgesics were converted to oral milligram morphine equivalents (MME). MME was calculated using standard MME conversion factors (https://pain.ucsf.edu/opioid-analgesics/calculation-oral-morphine-equivalents-ome).

### Statistical analysis

Analyses were performed with R software, version 3.6.3 (Foundation for Statistical Computing, Vienna, Austria). The features of the pain trajectory intercept and slope were calculated using an ordinary least squares fit regression equation based on the pain intensity of the 7 postoperative days. K-means clustering was used to specify all categories between 2 and 15, clustering the variables such as intercept and slope. The optimal number of clusters was determined based on the majority rule provided by the NbClust package in R.

The mean ± standard deviation was used to express normally distributed variables, while the median (interquartile range) was used for nonnormally distributed variables. Categorical data are described as numbers (percentages). Continuous variables among multiple groups were analysed using one-way ANOVA or the Kruskal–Wallis H test. Chi-square tests were performed to compare the categorical variables. A *P* value of < 0.05 was considered statistically significant. SAS 9.4 (SAS Institute Inc., Cary, NC) was used to perform multiple linear regression and multivariate logistic regression analyses.

## Results

**Figure 1:**
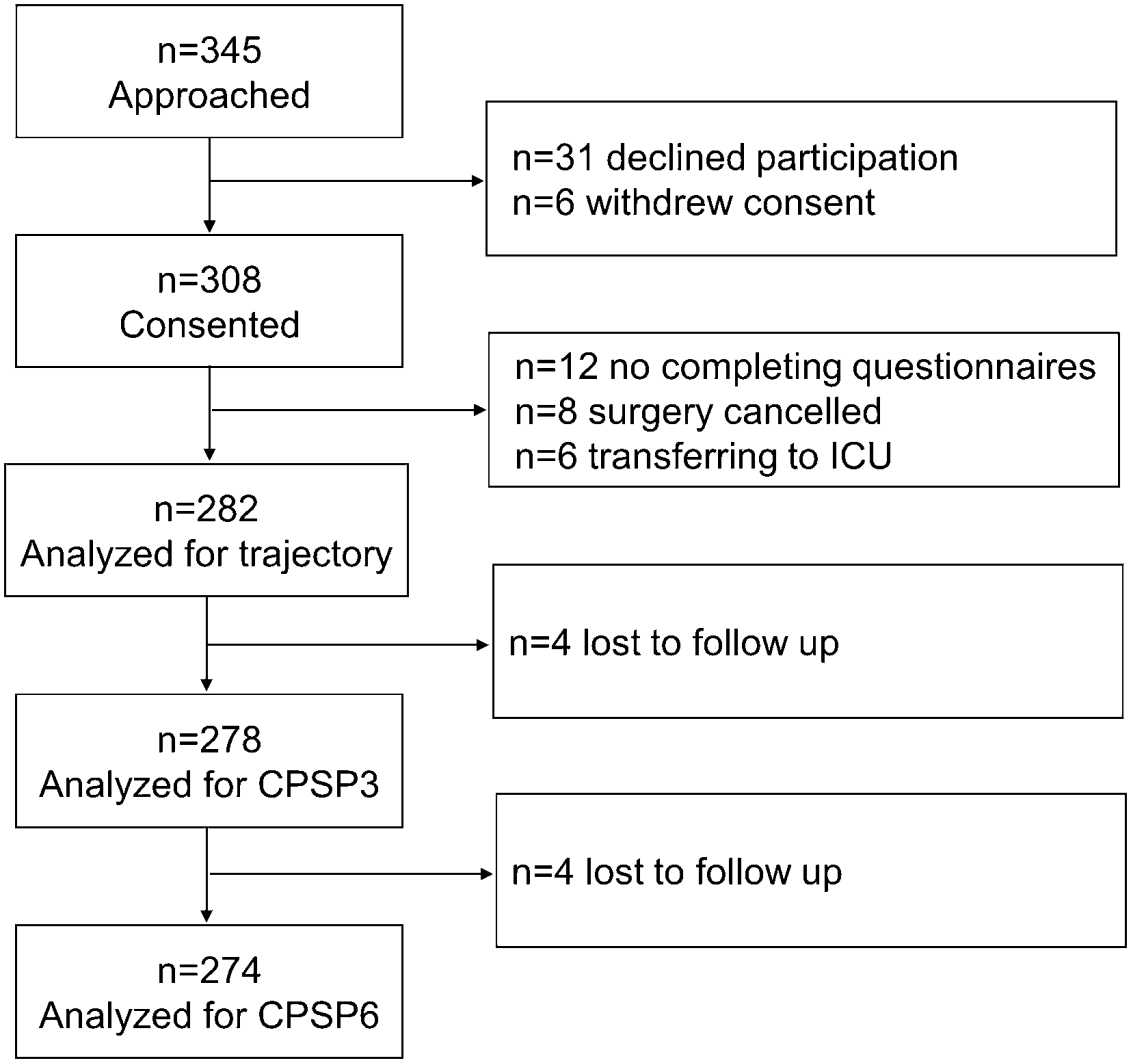
Flow diagram. *ICU* intensive care unit, *CPSP* Chronic postsurgical pain.

A total of 345 patients undergoing gastrointestinal surgery were recruited for this study. Of these, 31 refused to participate and 6 withdrew their consent. Excluded cases included 12 with incomplete questionnaires, 8 with surgical cancellation, and 6 with unplanned admission to the intensive care unit (ICU). A total of 282 patients (81.7%) were available for the analysis of APSP trajectory. Four and 8 patients were lost to follow-up within 3 and 6 months after surgery respectively (Fig. 1).

### Description and classification of APSP trajectory

**Figure 2:**
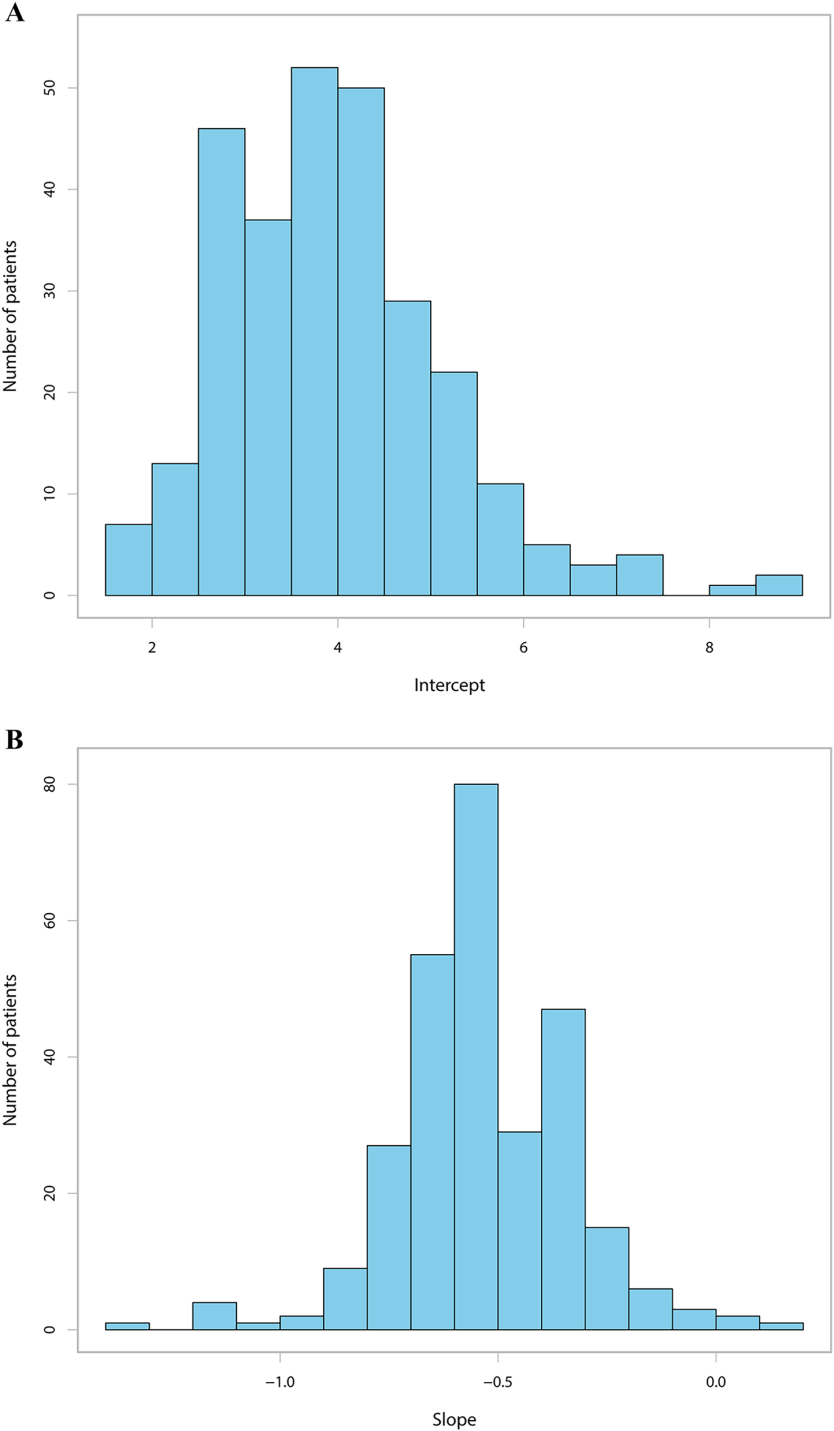
(**A**) Distribution of the APSP trajectory intercept. (**B**) Distribution of the APSP trajectory slope. *APSP* Acute postsurgical pain.

**Figure 3:**
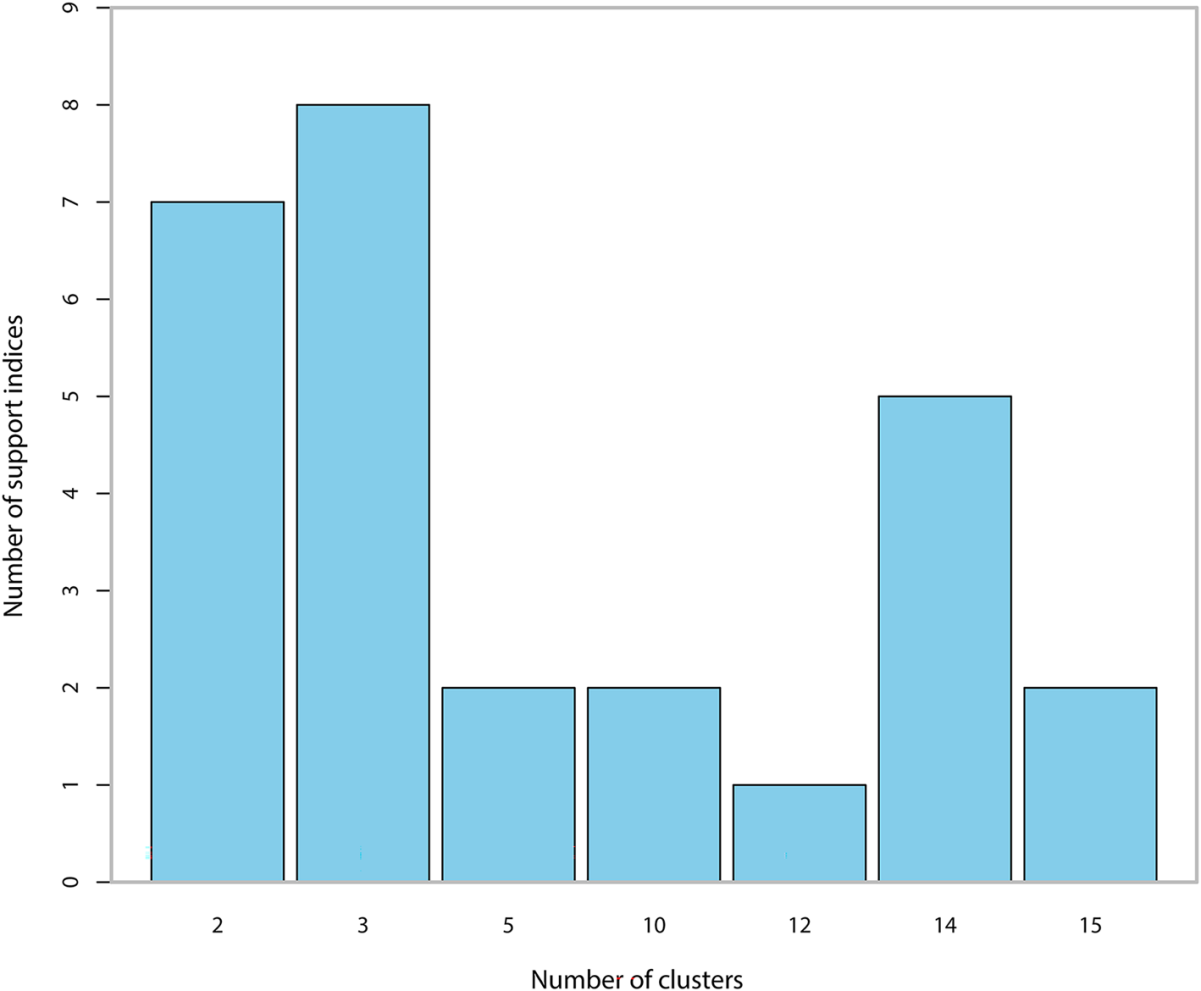
The optimal number of clusters. The optimal number of clusters were determined based on the majority rule provided by NbClust package.

**Figure 4:**
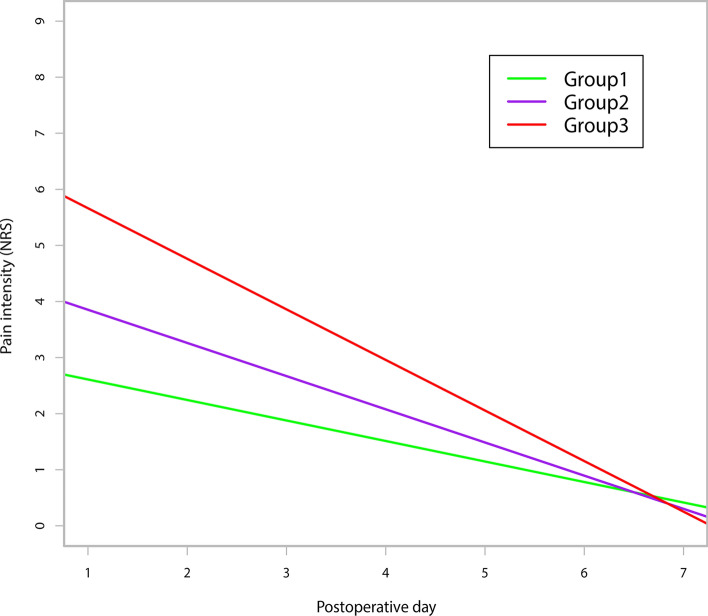
Results of the APSP trajectory analysis showing three different subgroups of patients. *APSP* Acute postsurgical pain.

The average APSP trajectory for the 282 patients is illustrated in Suppl. Figure 1. As shown in Suppl. Figure 2, the intercept was negatively correlated with the slope (Pearson’s r = −0.819, *P* ˂ 0.001). Figure 2A,B show the distribution of the intercept and the slope respectively. The majority of patients (73.7%) suffered moderate initial pain after surgery. Most patients had negative slopes, which demonstrated that pain was resolved gradually within 7 days. The K-means clustering algorithm shows that the optimal number of clusters is 3 (Fig. 3). Various indices according to built-in clustering scheme to determine the optimal cluster number are shown in Suppl. Table 1. The APSP trajectories of the three different groups are shown in Fig. 4. The initial pain intensity was the highest and resolved fastest over time in Group 3 (n = 24). The initial pain intensity was moderate and resolved more slowly in Group 2 (n = 141). In Group 1 (n = 117), the initial pain intensity was the lowest, and pain relief was the slowest.

Demographic, clinical, and psychological characteristics among the 3 trajectory groups are shown in Table 1. Three trajectories display negative slope. BMI (*P* = 0.0310), MME consumption (*P* = 0.0T337) and preoperative anxiety (*P* = 0.0448) were significantly different among the 3 groups.

**Table 1:** Demographic, clinical and psychological characteristics between 3 trajectory groups.

Characteristics	Group 1	Group 2	Group 3
	N = 117	N = 141	N = 24
**Gender**			
Male	71 (60.7%)	87 (61.7%)	14 (58.3%)
Female	46 (39.3%)	54 (38.3%)	10 (41.7%)
Age, year	66.6 ± 11.3	65.8 ± 11.7	62.2 ± 16.2
BMI, kg/m^2^	24.1 ± 4.6	25.7 ± 5.1	25.8 ± 5.7
**Occupation status**			
No occupation	94 (80.3%)	113 (80.1%)	18 (75.0%)
With occupation	23 (19.7%)	28 (19.9%)	6 (25.0%)
**Education level**			
Primary school or lower	40 (34.2%)	52 (36.9%)	7 (29.2%)
Middle or high school	56 (47.9%)	65 (46.1%)	14 (58.3%)
College or above	21 (17.9%)	24(17.0%)	3 (12.5%)
**Marital status**			
Single, separated or divorced	15 (12.8%)	21 (14.9%)	2 (8.9%)
Married	102 (87.2%)	120 (85.1%)	22 (91.7%)
**Smoking**			
Never	63 (53.9%)	82 (58.2%)	12 (50.0%)
Former	24 (20.5%)	28 (19.8%)	6 (25.0%)
Current	30 (25.6%)	31 (22.0%)	6 (25.0%)
**Alcohol drinking**			
Never	62 (53.0%)	82 (58.2%)	12 (50.0%)
Former	23 (19.7%)	35 (24.8%)	7 (29.12%)
Current	32 (27.3%)	24 (17.0%)	5 (20.8%)
Hypertension	62 (53.0%)	59 (41.8%)	10 (41.7%)
Diabetes	15 (12.8%)	20 (14.2%)	3 (12.5%)
Coronary heart disease	8 (6.8%)	14 (9.9%)	3 (12.5%)
**ASA physical status**			
ASA I	12 (10.3%)	27 (19.2%)	6 (25%)
ASA II	95 (81.2%)	93 (66.0%)	14 (58.3%)
ASA III	10 (8.6%)	21 (14.9%)	4 (16.7%)
Preoperative chronic pain	25 (21.4%)	33 (23.4%)	8 (33.3%)
Previous surgery	66 (56.4%)	63 (44.7%)	14 (58.3%)
**Site of surgery**			
Stomach	45 (38.5%)	62 (44.0%)	11 (45.8%)
Colorectum	64 (54.7%)	68 (48.2%)	13 (54.2%)
Small bowel	8 (6.8%))	11 (7.8%)	0 (0.00%)
**Type of surgery**			
Open	84 (71.8%)	95 (67.4%)	20 (83.3%)
Laparoscopic	33 (28.2%)	46 (32.6%)	4 (16.7%)
Duration of surgery, min	175.4 ± 60.1	183.4 ± 64.7	179.0 ± 53.5
MME consumption, mg	37.8 ± 15.8	45.7 ± 17.3	55.3 ± 18.2
**Number of drainage tubes**			
1	65 (55.6%)	78 (55.3%)	18 (75.0%)
≥ 2	52 (44.4%)	63 (44.7%)	6 (25.0%)
Tube retention time, day	7.6 ± 3.4	7.8 ± 2.6	9.1 ± 3.8
Length of hospital stay, day	19.5 ± 7.5	17.9 ± 5.9	20.7 ± 7.8
Malignant tumor	97 (82.9%)	119 (84.4%)	22 (91.7%)
HADS: anxiety	2.0 (1.0, 5.0)	2.0 (1.0, 7.0)	2.5 (1.5, 9.0)
Preoperative anxiety	18 (15.4%)	29 (20.6%)	9 (37.5%)
HADS: depression	2.0 (1.0, 5.0)	2.0 (1.0, 5.0)	2.5 (1.0, 6.5)
Preoperative depression	12 (10.3%)	21 (14.9%)	6 (25.0%)
Expected postsurgical pain intensity	5.0 (3.0, 6.0)	5.0 (3.0, 7.0)	6.0 (3.0, 7.0)
SFQ-s	8.0 (3.0, 14.0)	9.0 (3.0, 15.0)	10.5 (5.5, 25.5)
SFQ-l	6.0 (2.0, 14.0)	6.0 (1.0, 13.0)	8.0 (5.0, 20.0)

Data are presented as mean ± standard deviation, median (interquartile range), or number (percentage). *BMI* body mass index, *ASA* American Society of Anesthesiologists, *MME* morphine milligram equivalent, *HADS* Hospital Anxiety and Depression Scale, *SFQ-s* Surgical Fear Questionnaire short-time consequences, *SFQ-l* Surgical Fear Questionnaire long-time consequence.

### Predictors of the APSP trajectory intercept

In Suppl. Table 2, the univariate analysis shows that the APSP trajectory intercept was significantly associated with preoperative anxiety (β 0.09, 95% CI 0.05–0.12, *P* ˂ 0.001), preoperative depression (β 0.06, 95% CI 0.02–0.10, *P* = 0.0036), expected postsurgical pain (β 0.10, 95% CI 0.03–0.16, *P* = 0.0041) and MME consumption (β 0.06, 95% CI 0.04–0.08, *P* ˂ 0.001). In addition, age (β −0.13, 95% CI 0.25–0.02, *P* = 0.0252, by 10 years), BMI (β 0.05, 95% CI 0.02–0.07, *P* = 0.0013), preoperative chronic pain (β 0.51, 95% CI 0.18–0.84, *P* = 0.0024), SFQ-s (β 0.03, 95% CI 0.01–0.04, *P* = 0.0020) and SFQ-l (β 0.02, 95% CI 0.01–0.04, *P* = 0.0079) were correlated with the intercept.

A multiple linear regression analysis was conducted to determine the predictors of APSP trajectory intercept (Table 2). BMI (β 0.04 95% CI 0.01–0.07, *P* = 0.0067), preoperative chronic pain (β 0.40 95% CI 0.06–0.73, *P* = 0.0208), preoperative anxiety (β 0.12, 95% CI 0.08–0.15, *P* = 0.0305) and MME consumption (β 0.04, 95% CI 0.02–0.06, *P* = 0.0382) were independent predictors of the intercept.

**Table 2:** Multivariate analysis of predictors for APSP trajectory intercept.

Variables	β	95% CI	*P* value
BMI, kg/m^2^	0.04	0.01–0.07	0.0067
**BMI, kg/m**^**2**^			
≤ 25	Reference		
> 25, < 30	0.24	−0.37 to 0.42	0.9040
≥ 30	0.62	0.26–0.98	0.0010
**Preoperative chronic pain**			
No	Reference		
Yes	0.40	0.06–0.73	0.0208
MME consumption, mg	0.04	0.02–0.06	0.0382
HADS: anxiety	0.07	0.01–0.14	0.0305

*APSP* Acute presurgical pain*, BMI* body mass index, *MME* morphine milligram equivalent, *HADS* Hospital Anxiety and Depression Scale.

### Predictors of the APSP trajectory slope

Univariate logistic regression showed that the APSP trajectory slope was significantly correlated with age (OR 0.10, 95% CI 0.02–0.18, *P* = 0.0174, ≥ 65 vs. ≤ 50), BMI (OR −0.05, 95% CI −0.1 to −0.01, *P* = 0.0229), preoperative anxiety (OR −0.008, 95% CI −0.015 to −0.002, *P* = 0.0127) and MME consumption (OR −0.018, 95% CI −0.026 to −0.011, *P* = 0.0193) (Suppl. Table 3).

After adjustment for potential confounding factors, we observed that preoperative anxiety (OR −0.016, 95% CI −0.027 to −0.005, *P* = 0.0063) and MME consumption (OR −0.010, 95% CI −0.014 to −0.006, *P* = 0.0327) were independently associated with the slope (Table 3).

**Table 3:** Multivariate analysis of predictors for APSP trajectory slope. *APSP* Acute presurgical pain*, MME* morphine milligram equivalent, *HADS* Hospital Anxiety and Depression Scale.

Variables	β	95% CI	*P* value
MME consumption, mg	−0.010	−0.014 to −0.006	0.0327
HADS: anxiety	−0.016	−0.027 to −0.005	0.0063

### Predictors of CPSP at 3 months

A total of 278 patients completed follow-up at 3 months after surgery. The incidence of CPSP at 3 months was 30.6%. As shown in Suppl. Table 4, univariate analysis showed that the APSP trajectory (OR 0.56 95% CI 0.33–0.97, *P* = 0.0376, Group 2 vs. Group 1), preoperative anxiety (OR 1.09 95% CI 1.01–1.17, *P* = 0.0213), preoperative depression (OR 1.10 95% CI 1.02–1.17, *P* = 0.0128), SFQ-s (OR 1.03 95% CI 1.00–1.06, *P* = 0.0269) and SFQ-l (OR 1.04 95% CI 1.01–1.07, *P* = 0.0092) were significantly correlated with CPSP at 3 months. Meanwhile, CPSP at 3 months was correlated with female (OR 1.84 95% CI 1.09–3.08, *P* = 0.0215), age (OR 0.97 95% CI 0.95–0.99, *P* = 0.0123), preoperative chronic pain (OR 2.00 95% CI 1.13–3.56, *P* = 0.0178) and type of surgery (OR 0.53 95% CI 0.29–0.96, *P* = 0.0375).

After multivariable risk adjustment for potential confounding factors, the predictors of CPSP at 3 months included age (OR 0.93 95% CI 0.88–0.97, *P* = 0.0007) and APSP trajectory (OR 0.50 95% CI 0.26–0.93, *P* = 0.0297, Group 2 vs. Group 1) (Table 4).

**Table 4:** Multivariate analysis of predictors for CPSP at 3 months. *CPSP* Chronic postsurgical pain.

Variables	OR	95% CI	*P* value
Age, year	0.90	0.86–0.95	< 0.001
**Trajectory group**			
1	Reference		
2	0.45	0.23–0.89	0.0212
3	0.30	0.09–1.03	0.0558

Adjusted for gender, age, BMI, occupation status, education level, marital status, smoking, alcohol drinking, hypertension, diabetes, coronary heart disease, ASA physical status, preoperative chronic pain, previous surgery, site of surgery, type of surgery, duration of surgery, MME consumption, number of drainage tube, tube retention time ,length of hospital stay, malignant tumor, preoperative anxiety, preoperative depression, expected postsurgical pain intensity, SFQ-s, SFQ-l, and trajectory model.

### Predictors of CPSP at 6 months

A total of 274 patients completed follow-up at 6 months after surgery. The incidence of CPSP at 6 months was 16.4%. As shown in Suppl. Table 5, CPSP at 6 months was significantly correlated with preoperative anxiety (OR 4.05 95% CI 2.03–8.10, *P* < 0.001), preoperative depression (OR 1.09 95% CI 1.01–1.19, *P* = 0.0300) and SFQ-s (OR 1.04 95% CI 1.00–1.07, *P* = 0.0381). Additionally, female sex (OR 2.20 95% CI 1.15–4.20, *P* = 0.0170), age (OR 0.336 95% CI 0.13–0.87 *P* = 0.0253, ≥ 65 vs. ≤ 50) and preoperative chronic pain (OR 2.03 95% CI 1.02–4.03, *P* = 0.0439) were significantly correlated with CPSP at 6 months.

Multivariate logistic regression showed that female sex (OR 4.80 95% CI 1.16–19.91, *P* = 0.0305) and preoperative anxiety (OR 5.28 95% CI 1.59–17.54, *P* = 0.0067) were independent predictors of CPSP at 6 months (Table 5).

**Table 5:** Multivariate analysis of predictors for CPSP at 6 months. *CPSP* Chronic postsurgical pain.

Variables	OR	95% CI	*P value*
**Gender**			
Male	Reference		
Female	4.80	1.16–19.91	0.0305
**Preoperative anxiety**			
No	Reference		
Yes	5.28	1.59–17.54	0.0067

Adjusted for gender, age, BMI, occupation status, education level, marital status, smoking, alcohol drinking, hypertension, diabetes, coronary heart disease, ASA physical status, preoperative chronic pain, previous surgery, site of surgery, type of surgery, duration of surgery, MME consumption, number of drainage tube, tube retention time ,length of hospital stay, malignant tumor, preoperative anxiety, preoperative depression, expected postsurgical pain intensity, SFQ-s, SFQ-l, and trajectory model.

## Discussion

To the best of our knowledge, there were few prospective studies describing the APSP trajectory for gastrointestinal surgery, and very few studies have explored the association between the APSP trajectory and CPSP. In this study, we assessed possible predictors for the APSP trajectory for gastrointestinal surgery and identified the independent predictive effect of the APSP trajectory on CPSP. We discovered that BMI, preoperative chronic pain, preoperative anxiety, preoperative depression and MME consumption were independently associated with the APSP trajectory intercept. Meanwhile, preoperative anxiety and MME consumption were independently associated with the APSP trajectory slope. In addition, the study showed that age and APSP trajectory could independently predict CPSP at 3 months, and female sex and preoperative anxiety could independently predict CPSP at 6 months. However, the APSP trajectory could not predict CPSP at 3 months.

CPSP is strongly correlated with a high APSP trajectory intercept^18^. Sipilä et al.^19^ demonstrated that the predictors associated with the APSP trajectory intercept for breast cancer surgery were the amount of oxycodone needed for adequate analgesia and preoperative pain in the area of the operation. Additionally, a prospective cohort study described the APSP trajectory for mixed surgery and showed that the moderate and high pain trajectory group needed more postoperative opioids than the low pain trajectory group^20^. Similarly, our study found strong predictive factors for the intercept such as preoperative chronic pain and MME consumption. Meanwhile, we identified that preoperative anxiety was independently associated with the intercept. Althaus et al.^21^ investigated the predictive factors of the APSP trajectory and found that preoperative anxiety and female sex could independently predict the intercept. However, we did not find a predictive effect of age.

The APSP trajectory slope defined as resolution over time is strongly associated with potential factors. A prospective cohort study observed the risk factors for the acute pain trajectory after breast cancer and showen that BMI, expected postsurgical pain intensity and the amount of oxycodone needed for adequate analgesia were independent predictors of the APSP trajectory slope^19^. In our study, we found that MME consumption was independently associated with the slope. BMI was correlated with the slope but did not independently predict the slope after adjustment for potential confounding factors.

Preoperative anxiety was independently associated with APSP intensity and severity^22^. Pagé et al.^23^ demonstrated that preoperative anxiety could independently predict the APSP trajectory slope in a longitudinal study after total hip arthroplasty. A retrospective cohort study estimated the trajectory of postoperative pain using group-based trajectory modelling and discovered that preoperative anxiety and depression were predictors of the APSP trajectory slope^21^. Our study also showed that preoperative anxiety was an independent predictor of the slope, but preoperative depression was not.

APSP trajectory can dynamically reflect pain resolution over time, which contributes to identifying patients with risk factors for the development of persistent pain. In our study, trajectory Group 2, with the most favourable profile, was a protective factor for CPSP at 3 months.

The incidence of CPSP at 3 months in Group 2 was reduced by 55% compared with Group 1, which could probably be explained by the lower initial pain intensity and faster pain resolution in Group 2. In a prospective cohort study that investigated the acute pain trajectory after knee arthroplasty, the trajectory with low pain resolution was defined as an unfavourable pain trajectory. The study showed that an unfavourable maximal pain trajectory and pain trajectory at mobilization could independently predict the development of persistent pain^24^. Daoust et al.^11^ assessed the predictive power of acute pain intensity trajectories over 14 days after emergency department discharge for chronic pain 3 months later. They discovered that a trajectory with moderate initial pain intensity and flat slope was a strong risk factor for chronic pain 3 months later. Furthermore, Vasilopoulos et al.^20^ explored whether the APSP trajectory over 7 days after major surgery was a predictive factor for CPSP at 3 months. A pain trajectory with fast and sustained pain resolution was identified as an ideal pain trajectory. A nonideal pain trajectory was a trajectory with late, short, or no pain resolution. Their study showed that a nonideal pain trajectory was an independent predictor for CPSP at 3 months. Therefore, the pain trajectory with a flat slope was closely related to CPSP at 3 months.

In this study, however, we did not find a predictive effect for CPSP at 6 months. Pagé et al.^23^ investigated whether an acute movement-evoked postoperative pain trajectory over 5 days was associated with postsurgical pain 6 weeks and 6 months later. The pain trajectory with moderate initial pain intensity and a flat slope was identified as an unfavourable pain trajectory. Similar to our study, the unfavourable pain trajectory could only predict the development of postsurgical pain 6 weeks later, but could not predict the development of CPSP 6 months later. Therefore, in view of these studies, the unfavourable pain trajectory could predict the development of short-term postoperative pain instead of long-term postoperative pain.

Furthermore, we discovered that age and preoperative anxiety were predictive factors of CPSP at 6 months. Meanwhile, age was also a predictor of CPSP at 3 months. Similarly, Bruce et al.^25^ investigated the risk factors for chronic pain after gastrointestinal surgery and found that younger age and female sex were risk factors for CPSP. A population-based cross-sectional study showed that female sex was independently associated with chronic pain at 6 months after gastrointestinal surgery^26^. VanDenKerkhof et al.^27^ explored potential predictors for chronic pain after gastrointestinal surgery in a prospective single-centre cohort study. The results showed an independent association between preoperative anxiety and CPSP at 6 months. However, a retrospective study on laparoscopic colorectal surgery found that the predictors for CPSP at 6 months were not age, female sex or preoperative anxiety, but rather redo surgery for anastomotic leakage, inflammatory bowel disease and preoperative pain^28^. A possible reason was that psychological factors such as anxiety and depression were not included in the study. However, our study did not analyse several potential factors such as redo surgery and inflammatory bowel disease.

Our study had several methodological limitations. First, our study did not analyse preoperative pain sensitivity and genetic factors that were closely correlated with APSP and CPSP. Second, this study described the acute pain trajectory over 7 days. Therefore, the precision of repeated measurement data was probably reduced. In addition, these results cannot yet be generalised to the pain trajectory beyond the first 7 days after surgery. Finally, only 3- and 6-month follow-ups were conducted in our study. It is possible that patients suffer from persistent pain up to 1 year or longer after surgery. Further studies should investigate the association between the APSP trajectory and long-term pain outcomes.

## Conclusions

In conclusion, our results demonstrate the existence of three categories of APSP trajectories.

BMI, MME consumption, preoperative chronic pain and anxiety were predictors of the APSP trajectory intercept. Moreover, MME consumption and preoperative anxiety could independently predict the APSP trajectory slope. The APSP trajectory could predict the development of CPSP 3 months later, but not 6 months later. Moreover, age was a predictor of CPSP at 3 months, while female sex and preoperative anxiety could predict CPSP at 6 months. Clinicians need to stay alert for these predictors and pay close attention to pain resolution.

## Supplementary Information

Supplementary Information 1.

Supplementary Information 2.

## Data Availability

The datasets used and/or analysed during the current study are available from the corresponding author upon reasonable request.
