# Supplementary material for: Predictors and predictive effects of acute pain trajectories after gastrointestinal surgery
Source: Sci Rep. 2022 Apr 20;12:6530. doi: 10.1038/s41598-022-10504-5 (PMC9021210; doi:10.1038/s41598-022-10504-5)

Supplementary Figure

**Title:** Predictors and predictive effects of acute pain trajectories after gastrointestinal surgery

**Authors:** Qing-Ren Liu^1^, Yu-Chen Dai^2^, Mu-Huo Ji^3^, Li-Li Qiu^2^, Pan-Miao Liu^4^, Xing-Bing Sun^1^, Jian-Jun Yang^4﹡^

^1^Department of Anesthesiology, Xishan People’s Hospital of Wuxi City, Wuxi, 214105, China

^2^Department of Anesthesiology, Zhongda Hospital, Medical School, Southeast University,

Nanjing, 210009, China

^3^Department of Anesthesiology, The Second Affiliated Hospital, Nanjing Medical University, Nanjing, 210011, China

^4^Department of Anesthesiology, Pain and Perioperative Medicine, The First Affiliated Hospital of Zhengzhou University, Zhengzhou, 450000, China

^﹡^Correspondence: Jian-Jun Yang, NO. 1 East Jianshe Road, Zhengzhou, 450052, China

E-mail: yjyangjj@126.com

Suppl. Fig. 1. The APSP trajectory for the entire patients. *APSP* Acute postsurgical pain.


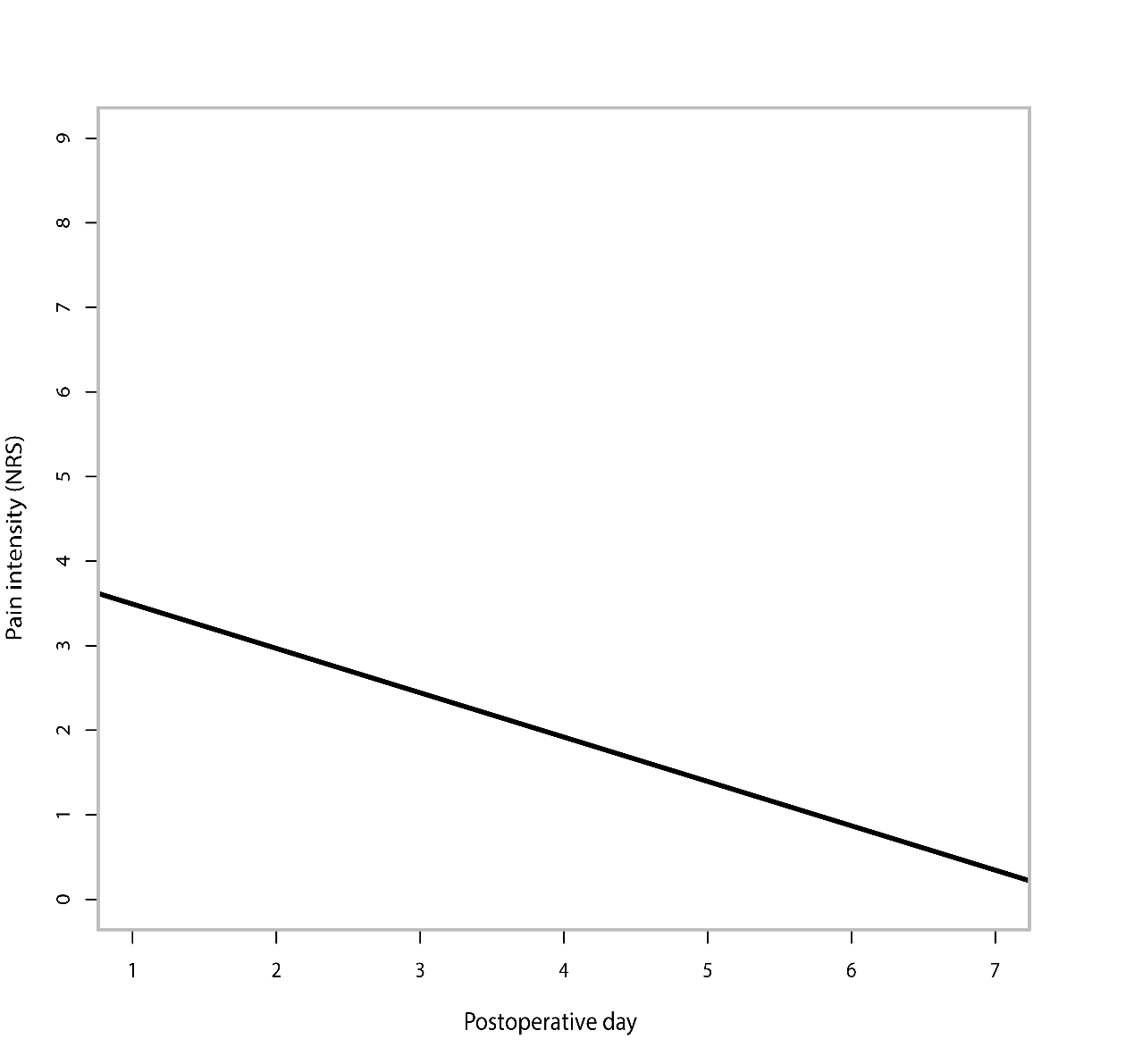


Suppl. Fig. 2. Jittered scatterplot showing the correlation between the intercept and the slope.


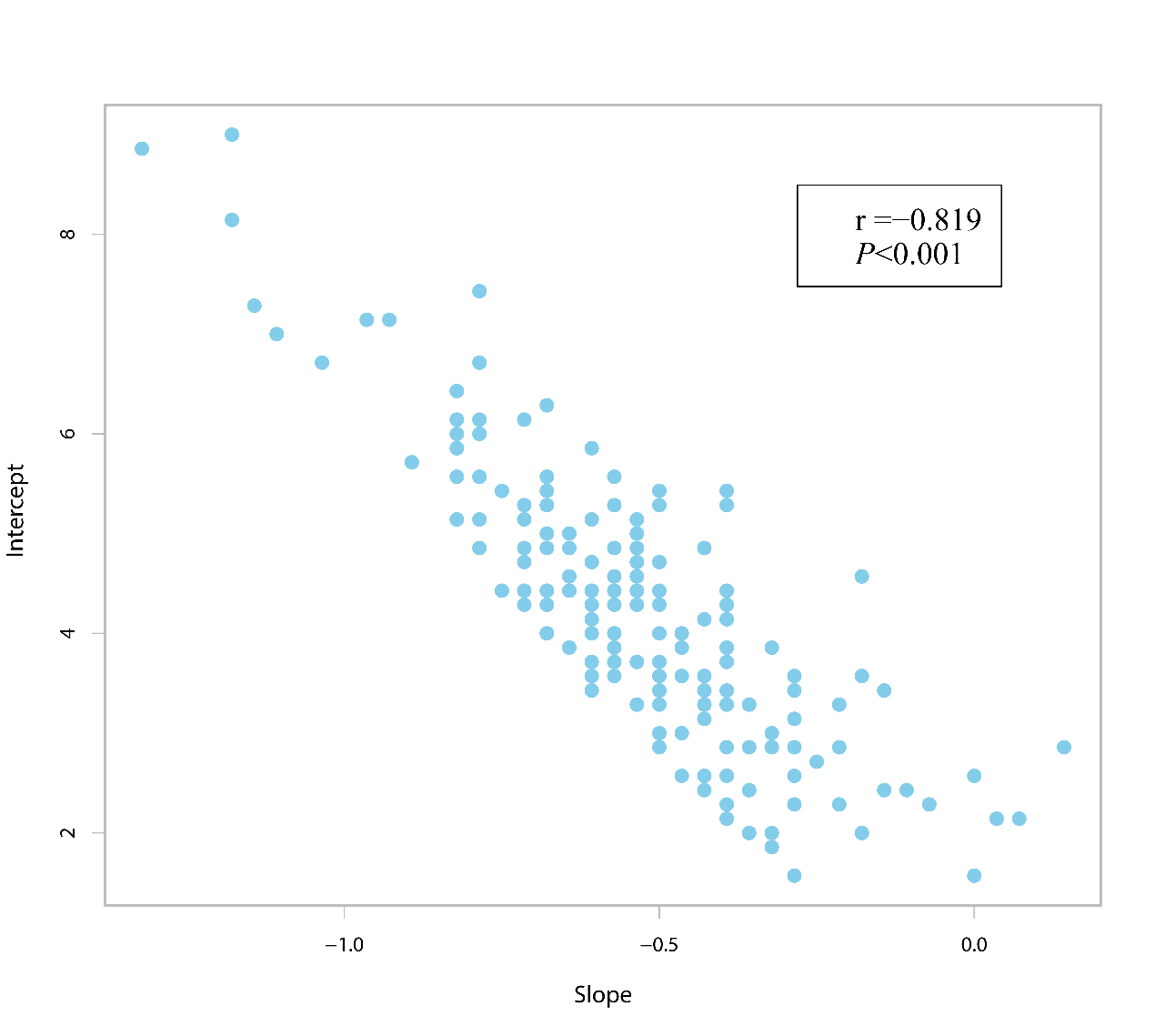

Supplement: Supplementary file 1 — Supplementary Information 1. [file 41598_2022_10504_MOESM1_ESM.docx]
