# Supplementary material for: Predictors and predictive effects of acute pain trajectories after gastrointestinal surgery
Source: Sci Rep. 2022 Apr 20;12:6530. doi: 10.1038/s41598-022-10504-5 (PMC9021210; doi:10.1038/s41598-022-10504-5)
Supplement: Supplementary file 2 — Supplementary Information 2. [file 41598_2022_10504_MOESM2_ESM.docx]

| Indices | Class 1 | Class 2 | Class 3 | Class 4 | Class 5 | Class 6 | Class 7 | Class 8 | Class 9 | Class 10 | Class 11 | Class 12 | Class 13 | Class 14 | Class 15 |
| --- | --- | --- | --- | --- | --- | --- | --- | --- | --- | --- | --- | --- | --- | --- | --- |
| KL | 0.52 | 3.00 | 0.53 | 2.35 | 3.41 | 1.60 | 0.17 | 10.83 | 1.63 | 0.41 | 1.17 | 0.20 | 7.60 | 1.74 | 3.00 |
| CH | 165.34 | 407.60 | 306.34 | 487.57 | 619.29 | 662.84 | 617.89 | 877.29 | 833.58 | 844.65 | 791.28 | 749.81 | 957.53 | 916.12 | 407.60 |
| Hartigan | 410.43 | 27.31 | 241.14 | 143.94 | 73.25 | 23.54 | 161.97 | 19.15 | 34.13 | 9.01 | 9.84 | 101.50 | 8.97 | 12.13 | 27.31 |
| CCC | 26.23 | 22.55 | 14.93 | 16.68 | 17.05 | 16.00 | 13.67 | 16.39 | 14.65 | 13.80 | 12.15 | 10.72 | 12.85 | 11.68 | 22.55 |
| Scott | 839.58 | 1100.06 | 1128.43 | 1314.22 | 1449.92 | 1529.31 | 1556.91 | 1716.13 | 1741.08 | 1789.47 | 1801.87 | 1834.59 | 1961.30 | 1990.06 | 1100.06 |
| Marriot | 3633.86 | 3246.33 | 5218.98 | 4219.65 | 3755.41 | 3857.24 | 4568.43 | 3287.49 | 3714.94 | 3786.30 | 4312.06 | 4506.34 | 3334.63 | 3456.98 | 3246.33 |
| TrCovW | 41891.98 | 6664.43 | 5462.04 | 1454.87 | 621.81 | 354.31 | 290.56 | 93.59 | 80.51 | 56.83 | 52.28 | 52.55 | 23.95 | 23.24 | 6664.43 |
| TraceW | 260.88 | 105.80 | 96.36 | 51.60 | 33.96 | 26.84 | 24.72 | 15.54 | 14.52 | 12.90 | 12.48 | 12.04 | 8.75 | 8.46 | 105.80 |
| Friedman | 64.08 | 95.86 | 99.64 | 146.85 | 219.92 | 258.16 | 275.93 | 440.32 | 478.75 | 541.47 | 564.14 | 580.19 | 888.18 | 892.89 | 95.86 |
| Rubin | 19.32 | 47.64 | 52.30 | 97.67 | 148.42 | 187.81 | 203.89 | 324.41 | 347.17 | 390.73 | 403.72 | 418.44 | 576.32 | 595.62 | 47.64 |
| Cindex | 0.25 | 0.24 | 0.27 | 0.28 | 0.27 | 0.29 | 0.35 | 0.26 | 0.30 | 0.30 | 0.26 | 0.26 | 0.21 | 0.21 | 0.24 |
| DB | 0.57 | 0.69 | 0.60 | 0.63 | 0.63 | 0.65 | 0.62 | 0.64 | 0.63 | 0.67 | 0.61 | 0.66 | 0.67 | 0.64 | 0.69 |
| Silhouette | 0.62 | 0.50 | 0.49 | 0.45 | 0.45 | 0.42 | 0.42 | 0.46 | 0.45 | 0.43 | 0.44 | 0.43 | 0.46 | 0.46 | 0.50 |
| Duda | 0.38 | 0.25 | 0.34 | 0.30 | 0.36 | 0.24 | 0.25 | 0.68 | 0.38 | 99.83 | 0.51 | 0.31 | 2.64 | 0.24 | 0.25 |
| Pseudot2 | 441.22 | 38.07 | 316.45 | 233.79 | 112.61 | 31.57 | 304.52 | 7.66 | 51.11 | -0.99 | 12.48 | 143.30 | -28.55 | 16.21 | 38.07 |
| Beale | 1.66 | 2.72 | 1.91 | 2.36 | 1.73 | 2.87 | 3.05 | 0.45 | 1.60 | -0.50 | 0.89 | 2.17 | -0.61 | 2.70 | 2.72 |
| Ratkowsky | 0.40 | 0.46 | 0.40 | 0.39 | 0.36 | 0.34 | 0.32 | 0.30 | 0.29 | 0.28 | 0.26 | 0.26 | 0.25 | 0.24 | 0.46 |
| Ball | 130.44 | 35.27 | 24.09 | 10.32 | 5.66 | 3.83 | 3.09 | 1.73 | 1.45 | 1.17 | 1.04 | 0.93 | 0.62 | 0.56 | 35.27 |
| Ptbiserial | 0.61 | 0.56 | 0.56 | 0.52 | 0.50 | 0.49 | 0.49 | 0.44 | 0.44 | 0.43 | 0.43 | 0.43 | 0.40 | 0.40 | 0.56 |
| Gap | 0.22 | 0.35 | -0.11 | 0.20 | 0.23 | 0.03 | -0.09 | 0.16 | -0.01 | -0.07 | -0.24 | -0.38 | -0.17 | -0.30 | 0.35 |
| Frey | 3.35 | -0.05 | 1.01 | 0.65 | 0.71 | 0.45 | 0.80 | 0.43 | 0.86 | 0.26 | 1.44 | 0.73 | 0.39 | 0.46 | -0.05 |
| McClain | 0.04 | 0.40 | 0.40 | 0.65 | 0.73 | 0.76 | 0.76 | 0.89 | 0.89 | 0.90 | 0.90 | 0.91 | 0.96 | 0.95 | 0.40 |
| Gamma | 0.85 | 0.71 | 0.71 | 0.79 | 0.84 | 0.86 | 0.86 | 0.91 | 0.92 | 0.92 | 0.92 | 0.92 | 0.95 | 0.95 | 0.71 |
| Gplus | 273.06 | 1431.31 | 1421.45 | 885.82 | 582.83 | 481.65 | 475.53 | 238.02 | 231.27 | 212.78 | 212.36 | 210.54 | 121.34 | 114.58 | 1431.31 |
| Tau | 5797.19 | NA | NA | NA | NA | NA | NA | NA | NA | NA | NA | NA | NA | NA | NA |
| Dunn | 0.03 | 0.05 | 0.05 | 0.08 | 0.09 | 0.11 | 0.14 | 0.14 | 0.16 | 0.17 | 0.18 | 0.19 | 0.20 | 0.22 | 0.05 |
| Hubert | 0.00 | 0.00 | 0.00 | 0.00 | 0.00 | 0.01 | 0.01 | 0.01 | 0.01 | 0.01 | 0.01 | 0.01 | 0.01 | 0.01 | 0.00 |
| SDindex | 5.95 | 4.47 | 3.10 | 3.53 | 4.12 | 4.62 | 4.36 | 5.97 | 6.01 | 7.01 | 6.57 | 8.63 | 9.02 | 9.09 | 4.47 |
| Dindex | 0.80 | 0.51 | 0.50 | 0.38 | 0.31 | 0.27 | 0.26 | 0.21 | 0.20 | 0.19 | 0.19 | 0.18 | 0.16 | 0.15 | 0.51 |
| SDbw | 0.96 | 0.91 | 0.45 | 0.52 | 0.35 | 0.41 | 0.20 | 0.11 | 0.15 | 0.11 | 0.02 | 0.03 | 0.02 | 0.02 | 0.91 |

**Suppl. Table 1.** Indices of group-based trajectory modelling. *KL* Kullback-Leibler, *CH* Calinski-Harabasz, *CCC* cubic clustering criterion, *DB* Davies-Bouldin.

| Variables | β | 95% CI | *P value* |
| --- | --- | --- | --- |
| Age，10 year | -0.13 | -0.25 -0.02 | **0.0252** |
| Age  ≤ 50  >50, <65  ≥ 65 | Reference  -0.58  -0.67 | -1.09 -0.06  -1.16 -0.18 | **0.0288**  **0.0081** |
| BMI, kg/m^2^ | 0.05 | 0.02 0.07 | **0.0013** |
| BMI, kg/m^2^  ≤ 25  > 25, < 30  ≥ 30 | Reference  0.12  0.66 | -0.27 0.51  0.31 1.02 | 0.5469  **0.0003** |
| Preoperative chronic pain  No  Yes | Reference  0.51 | 0.18 0.84 | **0.0024** |
| MME consumption, mg | 0.06 | 0.04 0.08 | **< 0.001** |
| HADS: anxiety | 0.09 | 0.05 0.12 | **< 0.001** |
| HADS: depression | 0.06 | 0.02 0.10 | **0.0036** |
| Expected postsurgical pain | 0.10 | 0.03 0.16 | **0.0041** |
| SFQ-s | 0.03 | 0.01 0.04 | **0.0020** |
| SFQ-l | 0.02 | 0.01 0.04 | **0.0079** |

**Suppl. Table 2.** Univariate analysis of predictors for APSP trajectory intercept. *APSP* Acute presurgical pain*, BMI* body mass index, *MME* morphine milligram equivalent, *HADS* Hospital Anxiety and Depression Scale, *SFQ-s* Surgical Fear Questionnaire short-time consequences, *SFQ-l* Surgical Fear Questionnaire long-time consequences.

| Variables | β | 95% CI | *P value* |
| --- | --- | --- | --- |
| Age，10 year | 0.01 | -0.0.1 0.03 | 0.2313 |
| Age  ≤ 50  >50, <65  ≥ 65 | Reference  0.12  0.10 | 0.03 0.20  0.02 0.18 | **0.0064**  **0.0174** |
| BMI, 10 kg/m^2^ | -0.05 | -0.1 -0.01 | **0.0229** |
| BMI, kg/m^2^  ≤ 25  > 25, < 30  ≥ 30 | Reference  -0.01  -0.07 | -0.08 0.05  -0.13 -0.01 | 0.6807  **0.0333** |
| MME consumption, mg | -0.018 | -0.026 -0.011 | **0.0193** |
| HADS: anxiety | -0.008 | -0.015 -0.002 | **0.0127** |

**Suppl. Table 3.** Univariate analysis of predictors for APSP trajectory slope. *APSP* Acute presurgical pain*, BMI* body mass index, *MME* morphine milligram equivalent, *HADS* Hospital Anxiety and Depression Scale.

| Variables | OR | 95% CI | *P value* |
| --- | --- | --- | --- |
| Gender  Male  Female | Reference 1.84 | 1.09 3.08 | **0.0215** |
| Age，year | 0.97 | 0.95 0.99 | **0.0123** |
| Preoperative chronic pain  No  Yes | Reference  2.00 | 1.13 3.56 | **0.0178** |
| Type of surgery  Open  Laparoscopic | Reference  0.53 | 0.29 0.96 | **0.0375** |
| HADS: anxiety | 1.09 | 1.01 1.17 | **0.0213** |
| HADS: depression | 1.10 | 1.02 1.17 | **0.0128** |
| SFQ-s | 1.03 | 1.00 1.06 | **0.0269** |
| SFQ-l | 1.04 | 1.01 1.07 | **0.0092** |
| Trajectory model  1  2  3 | Reference  0.56  1.04 | 0.33 0.97  0.42 2.59 | **0.0376**  0.9279 |

**Suppl. Table 4.** Univariate analysis of predictors for CPSP at 3 months. *CPSP* Chronic postsurgical pain*, HADS* Hospital Anxiety and Depression Scale.

| Variables | OR | 95% CI | *P value* |
| --- | --- | --- | --- |
| Gender  Male  Female | Reference 2.20 | 1.15 4.20 | **0.0170** |
| Age，year | 0.98 | 0.96 1.01 | 0.1665 |
| Age  ≤ 50  >50, <65  ≥ 65 | Reference  0.50  0.336 | 0.19 1.33  0.13 0.87 | 0.1637  **0.0253** |
| Preoperative chronic pain  No  Yes | Reference  2.03 | 1.02 4.03 | **0.0439** |
| HADS: anxiety | 1.13 | 1.04 1.23 | **0.0042** |
| Preoperative anxiety |  |  |  |
| No | Reference |  |  |
| Yes | 4.05 | 2.03 8.10 | **< 0.001** |
| HADS: depression | 1.09 | 1.01 1.19 | **0.0300** |
| Preoperative depression |  |  |  |
| No | Reference |  |  |
| Yes | 1.80 | 0.78 4.12 | 0.1676 |
| SFQ-s | 1.04 | 1.00 1.07 | **0.0381** |
| Trajectory model  1  2  3 | Reference  0.63  1.82 | 0.32 1.27  0.67 4.95 | 0.1964  0.2388 |

**Suppl. Table 5.** Univariate analysis of predictors for CPSP at 6 months. *CPSP* Chronic postsurgical pain*, HADS* Hospital Anxiety and Depression Scale, *SFQ-s* Surgical Fear Questionnaire short-time consequences.
